# Distal humerus prosthetic hemiarthroplasty: midterm results

**DOI:** 10.1007/s11751-015-0229-z

**Published:** 2015-08-27

**Authors:** Andras Heijink, Marc L. Wagener, Maarten J. de Vos, Denise Eygendaal

**Affiliations:** Department of Orthopaedic Surgery, Academic Medical Center (AMC), Meibergdreef 9, 1105 AZ Amsterdam, The Netherlands; Department of Orthopaedic Surgery, St. Radboud University Hospital, Nijmegen, The Netherlands; Department of Orthopaedic Surgery, Tergooi Hospitals, Hilversum, The Netherlands; Department of Orthopaedic Surgery, Amphia Hospital, Breda, The Netherlands

**Keywords:** Arthroplasty, Elbow, Posttraumatic, Trauma, Replacement, Upper extremity

## Abstract

Treatment of comminuted distal humeral fractures remains challenging. Open reduction–internal fixation remains the preferred treatment, but is not always feasible. In selected cases with non-reconstructable or highly comminuted fractures, total elbow arthroplasty has been used, however, also with relatively high complication and failure rates. Distal humerus prosthetic hemiarthroplasty (DHA) may be an alternative in these cases. The purpose of this study was to report the midterm results of six patients that were treated by DHA for acute and salvage treatment of non-reconstructable fractures of the distal humerus. All six patients were treated by DHA for acute and salvage treatment of non-reconstructable fractures of the distal humerus. Medical records were reviewed, and each patient was seen in the office. Mean follow-up was 54 months (range 21–76 months). Implant survival was 100 %. Three were pain free and three had mild or moderate residual pain. Average flexion–extension arc was 95.8° (range 70°–115°) and average pronation–supination arc was 165° (range 150°–180°). In three, there was some degree of instability, which was symptomatic in one. One had motoric and sensory sequelae of a partially recovered traumatic ulnar nerve lesion. According to the Mayo Elbow Performance Score, there were three excellent, one good and two poor results. Four were satisfied with the final result, and two were not. In this case series of six patients with DHA for non-reconstructable distal humerus fractures, favorable midterm follow-up results were seen; however, complications were also observed.

## Introduction

Treating comminuted distal humerus fractures remains challenging. Open reduction–internal fixation by means of double plating remains the gold standard, certainly in the young patient. However, the incidence of complications has been high, including reoperations and disappointing functional outcomes [1]. Also, in the elderly, results have been somewhat less predictable [1]. Some fractures are not amendable to open reduction–internal fixation (ORIF) due to the severity of comminution or poor bone quality. Total elbow arthroplasty (TEA) has been used as alternative treatment for non-reconstructable or severely comminuted fractures in the elderly [1]. Unfortunately, the incidence of complications after TEA has been relatively high [2]. This is even more so for posttraumatic indications [2]. Distal humerus prosthetic hemiarthroplasty (DHA) may be an alternative. DHA involves replacement of the distal humerus by a humeral component of a convertible total elbow system, mounted with an anatomical spool. Avoiding the ulnar component, loosening of which is responsible for large part of TEA failures, may reduce the complication rate as compared to TEA. Late conversion to total elbow arthroplasty is possible. First reported on in 1947, the earlier seven reports involving 28 cases with various indications date back to a time in which prosthetic material and design, understanding of elbow biomechanics, and surgical technique were less developed than they are today [3–9]. More recently, with advancement of understanding of elbow anatomy and biomechanics and the development of new prostheses, DHA has regained interest. Current expert opinion is that DHA may be considered for acute non-reconstructable fractures of the distal humerus or failed open reduction–internal fixation and/or posttraumatic sequelae of such fractures without realistic reconstruction options (e.g., nonunion, avascular necrosis). Some include fracture types that are generally associated with complete disruption of the vascularization of the distal fragments (e.g., coronal shear fracture of the capitellum and lateral trochlea combined with low transverse bicondylar extension). The decision as to when a fracture is considered non-reconstructable depends on fracture characteristics, bone quality and surgeon experience. To date, clinical data related to DHA using modern era prostheses and surgical principles are limited to eight peer-reviewed publications reporting on six series including 60 cases (Table 1) [10–17]. Forty-nine of those cases involved treatment of acute fractures, and 11 were salvage procedures (i.e., reconstruction after failed open reduction–internal fixation). Three commercial implant systems were used: the Sorbie-Questor total elbow prosthesis (Wright Medical, Arlington, TN, USA), the Kudo total elbow prosthesis (Biomet, Warsaw, IN, USA) and the Latitude Total Elbow Prosthesis (Tornier, Montbonnot, France).

**Table 1:** Summary of recent literature

References	Study design	*N*	Average follow-up	Prosthesis type	Data	Implant failure (%)	Average postoperative flexion–extension arc (°)	MEPS	Degeneration proximal ulna leading to (planned) conversion
Parsons et al. [15]	Case series	8 (4 acute, 4 salvage)	Not provided, short-term	Sorbie-Questor	Pooled	100	Not provided	Not provided	1
Burkhart et al. [13, 14]	Case series	10 (8 acute, 2 salvage)	12.1 (6–23) months	Latitude	Individualized	100	107 (range 75–135)	8 excellent, 1 good, 1 fair	1
Adolfsson et al. [10, 11]	Case series	8 (all acute)	4.1 (range 2.5–6) years	Kudo	Individualized	100	96.3 (range 60–120)	5 excellent, 3 good	None
Argintar et al. [12]	Case series	10 (9 acute, 1 salvage)	12 months (range not provided)	Latitude	Individualized	100	102 (range 110–140)	3 excellent, 2 good, 3 fair, 1 poor, 1 n/a	None
Smith et al. [17]	Case series	17 (15 acute, 2 salvage)^a^	80 (range 25–133) months	5 Sorbie-Questor, 12 Latitude	Individualized	15 %^b^	116 (range 70–133)	4 excellent, 4 good, 1 fair, 1 poor	None
Hohman et al. [16]	Case series	7 (5 acute, 2 salvage)^c^	36 months (range not provided)	Latitude	Individualized	100	96 (range 70–130)	1 excellent, 3 good, 2 fair, 1 poor	None

^a^Four revised prostheses were not included in the review, and four patients had died. One was lost to follow-up for reasons not discussed

^b^Implant failure was calculated based on four failures/revisions of a total of 26 placed prostheses, assuming the nine prosthesis that were lost to follow-up have not failed

^c^One patient was lost to follow-up

The midterm follow-up results of six patients that were treated by distal humerus prosthetic hemiarthroplasty for non-reconstructable fracture of the distal humerus or failure of ORIF of such fractures are reported.

## Materials and methods

Approval for this study was waived from our institutions’ Medical Ethical Committee, and each patient was informed that data concerning their case would be submitted for publication.

Six patients were treated in our institution by distal humerus prosthetic hemiarthroplasty (DHA) between April 2006 and November 2009: One was treated for a closed non-reconstructable fracture of the distal humerus, while five were treated for failed earlier treatment and/or sequelae of such a fracture without realistic reconstruction options.

During surgery, the patient was in the lateral decubitus position with the arm in a support and flexed to 90°, the humerus parallel and forearm perpendicular to the floor. Prophylactic antibiotic coverage consisted of 2 g cefazolin intravenously. A tourniquet was used. An (extensile) posterior approach was carried out, during which both epicondyles were exposed and the ulnar nerve was identified and mobilized, but not anteriorly transposed. In three patients (cases 3, 4 and 5), an apex-distal chevron olecranon osteotomy was performed at the bare area of the sigmoid fossa and the triceps mechanism was reflected sufficiently proximally to expose the distal humerus. In three others (cases 1, 2 and 6), the ulnohumeral joint was dislocated after subperiosteal release of the lateral collateral ligament complex. The comminuted articular segments were then removed, taking care to preserve the medial and lateral epicondyles and to protect the origin of the collateral ligament. The distal humeral articular segments, the radial head and coronoid were used as templates for choosing the correct implant size. The superior aspect of the olecranon fossa was resected, and the humeral canal was broached and reamed. A trial component was then placed, using local landmarks such as the insertion of the collateral ligaments and the condyles to determine adequate depth. With the trial prosthesis in situ, the elbow was reduced and range of motion and stability were tested. Subsequently, the final prosthesis was cemented in place. The olecranon osteotomy was repaired using a tension band technique consisting of a large screw 6.0 mm in diameter and an Orthocord^®^ (Biomet, Warsaw, IN, USA) tension band. Alternatively, the lateral collateral ligament was reconstructed using transosseous sutures. A removable splint for the night was provided for 6 weeks to allow proper soft tissue healing. Passive range of motion started on the first postoperative day, and active range of motion was resumed after 6 weeks, both under the supervision of a physical therapist. All procedures were performed by two senior shoulder and elbow surgeons (D.E. and M.V.).

Medical records were reviewed, and each patient was seen in the office for a clinical assessment and radiographic evaluation. At the office, range of motion was measured using a goniometer and instability was tested for in extension and 30° of flexion by the moving valgus stress test. Instability was graded none (grade 0), medial tenderness with valgus stress (grade 1), mild instability (grade 2) or subluxation (grade 3). Elbow function was further assessed using the Mayo Elbow Performance Score [18, 19] and Oxford Elbow Score (OES). In addition, the Disabilities of Arm, Shoulder and Hand (DASH) questionnaire and Short Form (SF)-36 questionnaire were administered. For one patient that had died of natural causes (case 5), documentation from the last clinic visit was used. Radiographs of the elbow were reviewed for signs of implant loosening, degenerative changes of the ulnar trochlea and periarticular heterotopic ossifications. Periarticular heterotopic ossifications were graded as previously described by Brooker et al. [20].

**Fig. 1:**
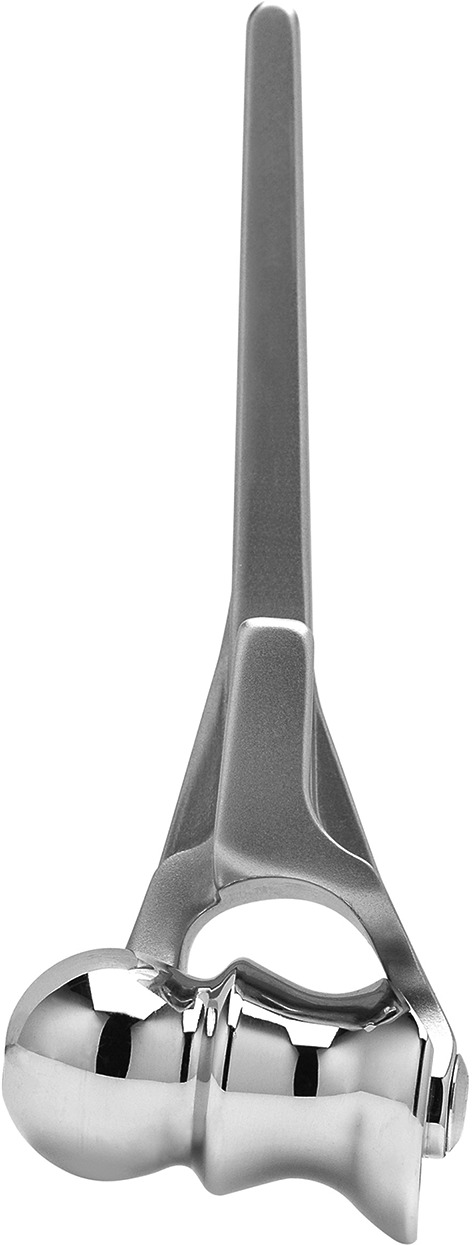
Humeral component with anatomical spool (i.e., distal humerus prosthesis) of the Lattitude^®^ Total Elbow

All six patients were females. There were four right and two left and four dominant elbows involved. Mean age at surgery was 69 (range 55–77 years). In one patient, treatment was for acute fracture (case 5), in one for avascular necrosis of the capitellum (case 2) and in four for symptomatic nonunion. None had open wounds or impaired skin or soft tissues. None had degenerative changes of the proximal ulna on the preoperative imaging studies. In all patients, the humeral component of the Latitude^®^ Total Elbow Prosthesis (Tornier, Montbonnot, France) was used with an anatomical spool (Fig. 1). All were cemented in place and inserted according to the manufacturer’s recommendations. Mean follow-up was 54 months (range 21–76 months).

## Results

Demographic data, overall clinical outcome data and patient-derived outcome data of this patient sample are presented (Tables 2, 3, 4, respectively). Three patients had no pain, two had mild, and one had moderate pain. Four were satisfied and two were dissatisfied with the final result. Average flexion was 122.5° (range 110°–130°), average extension deficit was 26.7° (range 20°–40°), and the average flexion–extension arc was 95.8° (range 70°–115°). Average pronation was 84.2° (range 75°–90°), average supination was 80.1° (range 75°–90°), and the average pronation–supination arc was 165° (range 150°–180°). One patient had grade 1 (case 1), one patient had grade 2 (case 3), and one patient had grade 3 (case 2) valgus instability with testing. The patient with grade 3 instability also complained of subjective instability during activities of daily living. Interestingly, two of those three patients with valgus instability (cases 1 and 2) had had a subperiosteal release and subsequent reattachment of the lateral collateral ligament complex, and both were also the patients that were unsatisfied with the final outcome. According to the Mayo Elbow Performance Score, there were three excellent, one good and two poor results. There were two neurovascular complications. One patient had motoric and sensory sequelae of an EMG-proven axonotmesis of the ulnar nerve that has only partially recovered to date. Another had decreased sensation in the ulnar nerve distribution preoperatively that had fully recovered at end follow-up. There were no infections and no systemic complications.

**Table 2:** Demographic data for the individual patients

Case	Sex	Injured side	Dominant side	Age at surgery	AO classification initial fracture	Indication	Surgical procedures prior to distal humerus hemiarthroplasty	Time from initial fracture treatment (months)
1	Female	Right	Right	62	13 type b2	Nonunion capitellum w/secondary avascular necrosis	ORIF	29
2	Female	Right	Right	55	13 type c3	Severe avascular necrosis capitellum	ORIF	10
3	Female	Right	Right	77	13 type b3	Nonunion capitellum w/secondary avascular necrosis	ORIF	1
4	Female	Left	Right	65	13 type b3	Nonunion capitellum w/secondary avascular necrosis	ORIF	4
5	Female	Right	Right	76	13 type c3	Acute non-reconstructable distal humerus fracture	None	0
6	Female	Left	Right	68	13 type b3	Nonunion capitellum w/secondary avascular necrosis capitellum	ORIF	7

*ORIF* open reduction–internal fixation

**Table 3:** Clinical outcome data for the individual patients

Case	F/U (mos)	Pain^a^	Instability^b^	Flexion–extension (arc) (zero method, °)	Pronation–supination (arc) (zero method, °)	Mayo Elbow Performance Score^c^	Neurovascular or infectious complications	Additional comments	Patient satisfaction
1	76	Mild	Grade 1 valgus	110–40–0 (70)	75–0–75 (150)	55/poor	Fully recovered lesion ulnar nerve not neurophysiologically investigated	None	Unsatisfied
2	61	Moderate	Grade 3 valgus	115–30–0 (85)	90–0–80 (170)	40/poor	Partially recovered EMG-proven axonotmesis ulnar nerve	Persistent subluxation	Unsatisfied
3	57	Mild	Grade 2 valgus	130–30–0 (100)	90–0–90 (180)	80/good	None	None	Satisfied
4	66	None	None	135–20–0 (115)	80–0–80 (160)	100/excellent	None	None	Satisfied
5^d^	21	None	None	115–20–0 (95)	80–0–70 (150)	95/excellent	None	None	Satisfied
6	43	None	None	130–20–0 (110)	90–0–90 (180)	100/excellent	None	None	Satisfied

^a^Pain is graded as none, mild, moderate or severe

^b^Instability is graded as none (i.e., stable), mild, moderate or gross

^c^The Mayo Elbow Performance Score total score is graded as excellent (95–100), good (80–94), fair (60–79) and poor (<59). All revisions are considered a poor result, regardless of total score

^d^Patient deceased due to old age. Documentation from last office visit was used

**Table 4:** Patient-derived outcome scores

Case	DASH	Oxford Elbow Score			SF-36	
		Pain domain	Elbow function	Socio-psychological	Physical component summary	Mental component summary
1	20	100	68.8	50	43.6	49.1
2	57.5	43.8	25	31.3	37.5	51.5
3	7.4	100	100	87.5	47.8	56.4
4	5.0	87.5	87.5	93.8	54.3	39.4
5	n/a	n/a	n/a	n/a	n/a	n/a
6	2.5	87.5	100	87.5	53.9	59.5

**Fig. 2:**
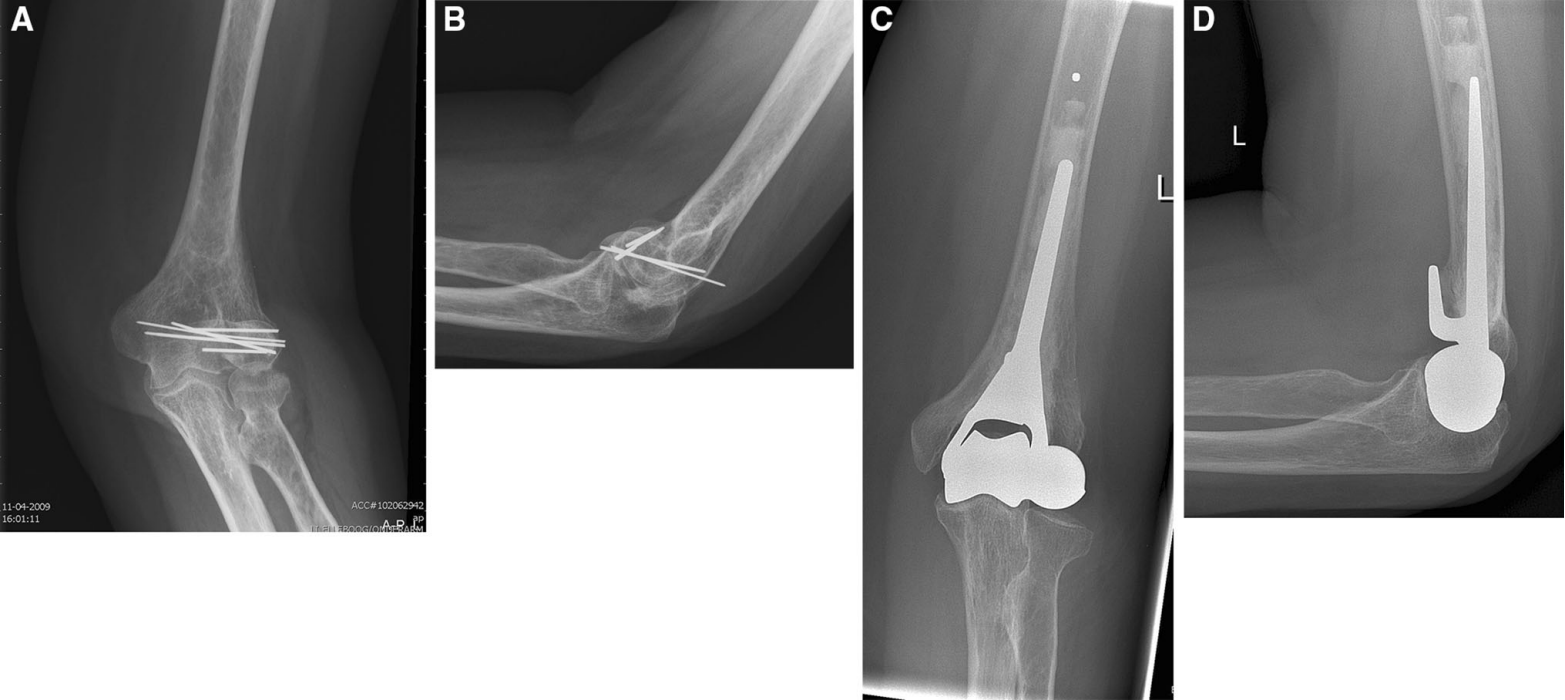
**a**–**d** Antero-posterior and lateral radiographic images of the elbow before (**a**, **b**) and at end follow-up (**c**, **d**) 43 months after distal humerus prosthetic hemiarthroplasty (humeral component of Latitude^®^ Total Elbow with anatomical spool) at end follow-up of case 6. The prosthesis is well positioned, without signs of loosening

**Fig. 3:**
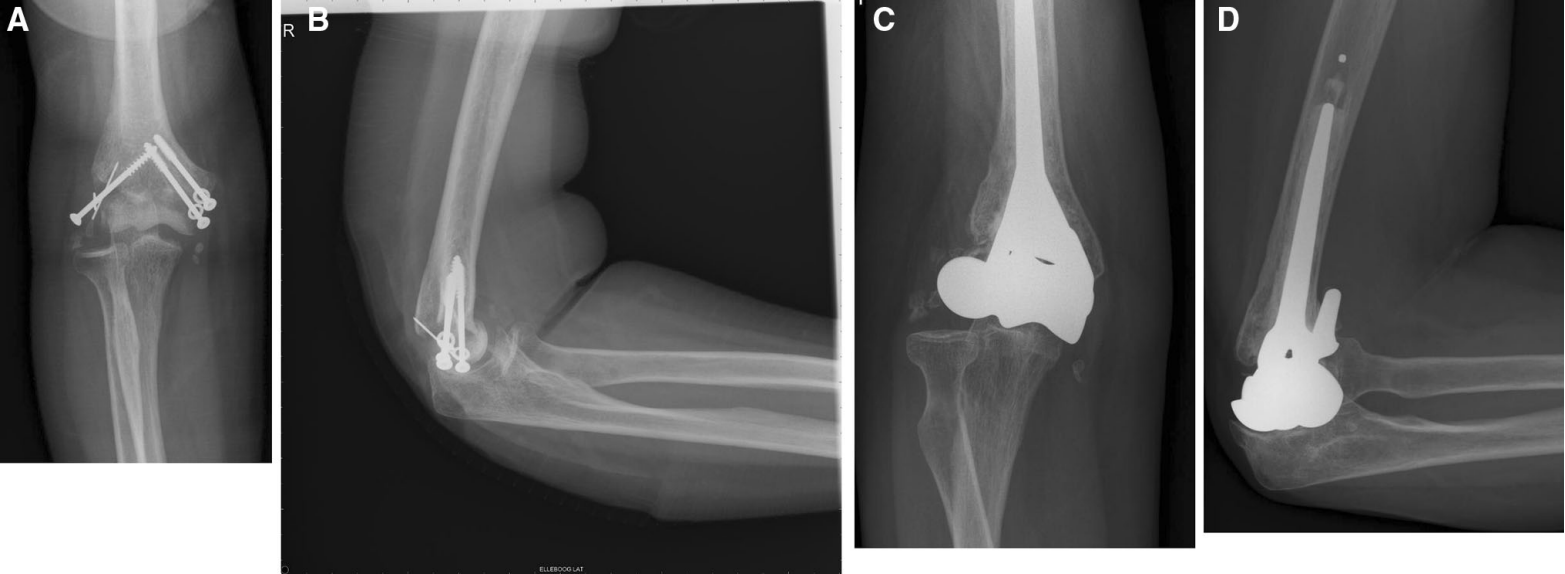
**a**–**d** Antero-posterior and lateral radiographic image of the elbow before (**a**, **b**) and at end follow-up (**c**, **d**) 61 months after distal humerus prosthetic hemiarthroplasty (humeral component of Latitude^®^ Total Elbow with anatomical spool) of case 2. Subluxations of the ulnohumeral joint and discrete irregularity of the articulating surface of the proximal ulna are noted

At end follow-up, implant survival was 100 % with no radiographic signs of loosening in any case (case 6, Fig. 2a–d). In one patient (case 2), there was subluxation of the ulnohumeral joint with slight radiographic attrition of the proximal ulna (case 2, Fig. 3a–d). This was the same patient with grade 3 valgus instability. Heterotopic ossifications (Brooker II) had developed in one patient (case 5). Two patients had developed calcifications related to reconstruction of the lateral collateral ligament (cases 1 and 2). None of the others showed erosion of the proximal ulna.

## Discussion

Distal humerus hemiarthroplasty (DHA) involves replacement of the distal humerus by a humeral component of a convertible total elbow system, mounted with an anatomical spool. The midterm follow-up results of six consecutive cases were reported.

Terminology has been variable and mostly not discerning well-enough isolated distal humerus replacement from total elbow replacement. We suggest using the term *distal humerus prosthetic hemiarthroplasty* in future when describing this procedure, as it is (1) descriptive of the procedure, (2) avoids confusion with radiocapitellar prosthetic arthroplasty, which has also been referred to as a hemiarthroplasty and (3) avoids confusion with other types of arthroplasties, such as resection arthroplasty, interposition arthroplasty.

Anatomical prerequisites for DHA are (1) an intact or stable radial head, (2) an intact or reconstructable coronoid process, (3) intact or reconstructable columns, (4) a presumably stable (i.e., ligamentous intact) elbow or reconstructable collateral ligaments and (5) absence of ulnohumeral degenerative changes. Patients should also be too young for TEA. DHA is considered an *‘unlinked’* construction and therefore must mimic the native distal humerus anatomy in order to provide optimal stability and transmission of forces. The implant must be inserted at the correct level and orientation to restore the axis of flexion in relation to the insertions of the medial and lateral collateral ligaments.

Our observations made in and conclusions drawn from this study are obviously only as strong and valid as can be with a case series. However, cases series are still the only and therefore *best available* evidence to date on this topic and will probably remain so for quite some time.

Over all, our results compare well with the current literature [10–17]. Smith et al. reported 4 failures of 26 placed prostheses (15 %, assuming the nine prostheses that were lost to follow-up have not failed) due to periprosthetic fracture and loosening. All other authors reported 100 % implant survival, although both Parsons and Burkhart had scheduled a conversion to total elbow arthroplasty (TEA) for symptomatic ulnar wear. In the current series, no implants failed. None of the authors reports an objective measurement of residual pain, and only Hohman et al. comment on patient satisfaction by reporting a mean Likert satisfaction scale of 7 of 10. Comparison with the current series, in which two out of six patients (33 %) were unsatisfied with the final outcome, is therefore not possible. Our results expressed in terms of Mayo Elbow Performance Score (MEPS) are with 67 % combined excellent or good results mid-range when compared to the current literature (range 50–100 %), as is the range of ulnohumeral motion. Complications are not all that uncommon, some minor, but some more serious. Hohman et al. reported one peroperative diaphyseal humerus fracture and Argintar et al. one peroperative olecranon fracture, both successfully treated by plate fixation. As stated, Smith et al. reported two periprosthetic fractures for which conversion to total elbow arthroplasty was performed, respectively, 54 and 140 months after placement of the prosthesis. Adolfsson et al. observed one periprosthetic fracture 3 years after placement of the prosthesis, which was successfully treated by plate fixation. Smith et al. also revised 2 of 26 placed prostheses (8 %, assuming the nine prostheses that were lost to follow-up have not failed) for loosening. All but one author reported ulnar nerve neuropathy, either transient or persistent, requiring transposition, mostly in around 10 % of the cases, but for Smith et al. even in up to 4 of 17 (24 %). In the current series, two of six patients (33 %) had ulnar nerve problems, one a transient neuropathy, the second a not recovered axonotmesis, which was sustained during the trauma. We appear to be first to encounter valgus instability, which will be discussed in the next paragraph. Wound problems or infection were uncommon, as were nonunion of the olecranon osteotomy. Frequently, Smith et al. reported in up to 59 % of reviewed patients, symptomatic hardware, used to fix the olecranon osteotomy, had to be removed. Smith et al. reported one case of elbow stiffness, requiring arthrolysis. Radiographically, all but one author reported wear of the proximal ulna to some extent. Parsons et al. had scheduled one of eight (12.5 %) patients for conversion for the same reason at not specified short-term follow-up for symptomatic ulnar wear and Burkhart one of 10 (10 %) after 13-month follow-up. Further, Adolfsson et al. observed attrition of the proximal ulna in three of eight (37.5 %) that was only mildly symptomatic at most, which could already be noticed at 2 years in two patients and after 6 years in three. Smith et al. reported ulnar wear in 13 of 17 (76 %) reviewed patients: grade 1 (partial-thickness cartilage loss) in 7, grade 2 (full-thickness cartilage loss) in 4 and grade 3 (bone loss) in 2. Hohman et al. report ulnar wear in all seven patients: mild (preserved joint space) in three, moderate (loss of joint space, but no bone loss) in two and severe (significant bone loss) in two. In the current series, the one patient with the subluxed ulnohumeral joint had attrition of the proximal ulna, but the other did not at midterm follow-up.

From the current series, an important observation can be made. Two out of three patients in whom the lateral collateral ligament was released to dislocate the joint without olecranon osteotomy (cases 1 and 2) were unsatisfied with the final outcome. The first patient has mild residual pain and mild (grade 1) valgus instability. The second (case 2) has moderate residual pain gross (grade 3) valgus instability and a radiographically subluxed prosthesis. In the first case (case 1), possibly the stress on the medial ulnar collateral ligament (MUCL) during the period of dislocation was too great that it resulted in persistent insufficiency of the ligament. In the second case (case 2), it seems the reattachment of the lateral ligament complex has failed. Although being unsatisfied with the final outcome, this patient refused further surgery. We therefore feel very strongly that release of the lateral collateral ligament should not routinely be performed, and we prefer an olecranon osteotomy for exposure and dislocation of the joint without ligamentous release.

Another observation is that salvage procedures, as is the case for five of six cases in the current series, show comparable results and complication ratios as acute cases, of which the majority (82 %) of data of the current literature are derived from [10–17].

In this case series of six patients with DHA for non-reconstructable distal humerus fracture, favorable midterm follow-up results were seen, albeit fair to say that two patients are not satisfied with the final outcome. We feel very strongly that release of the lateral collateral ligament should not routinely be performed and would advise an olecranon osteotomy without ligamentous release to be performed for exposure and dislocation of the joint. The role of DHA in acute fracture care is not well defined to date, but the technique may increasingly need to be considered with the increasing incidence of complex, osteopenic fractures in the elderly.

## References

[1] McKee MD, Veillette CJ, Hall JA, Schemitsch EH, Wild LM, McCormack R, Perey B, Goetz T, Zomar M, Moon K, Mandel S, Petit S, Guy P, Leung I (2009). A multicenter, prospective, randomized, controlled trial of open reduction—internal fixation versus total elbow arthroplasty for displaced intra-articular distal humeral fractures in elderly patients. J Shoulder Elbow Surg.

[2] Voloshin I, Schippert DW, Kakar S, Kaye EK, Morrey BF (2011). Complications of total elbow replacement: a systematic review. J Shoulder Elbow Surg.

[3] Barr JS, Eaton RG (1965). Elbow reconstruction with a new prosthesis to replace the distal end of the humerus. A case report. J Bone Joint Surg Am.

[4] Macausland WR (1947). Arthroplasty of the elbow. N Engl J Med.

[5] Mellen RH, Phalen GS (1947). Arthroplasty of the elbow by replacement of the distal portion of the humerus with an acrylic prosthesis. J Bone Joint Surg Am.

[6] Shifrin PG, Johnson DP (1990). Elbow hemiarthroplasty with 20-year follow-up study. A case report and literature review. Clin Orthop Relat Res.

[7] Street DM, Stevens PS (1974). A humeral replacement prosthesis for the elbow: results in ten elbows. J Bone Joint Surg Am.

[8] Swoboda B, Scott RD (1999). Humeral hemiarthroplasty of the elbow joint in young patients with rheumatoid arthritis: a report on 7 arthroplasties. J Arthroplasty.

[9] Venable CS (1952). An elbow and an elbow prosthesis; case of complete loss of the lower third of the humerus. Am J Surg.

[10] Adolfsson L, Hammer R (2006). Elbow hemiarthroplasty for acute reconstruction of intraarticular distal humerus fractures: a preliminary report involving 4 patients. Acta Orthop.

[11] Adolfsson L, Nestorson J (2012). The Kudo humeral component as primary hemiarthroplasty in distal humeral fractures. J Shoulder Elbow Surg.

[12] Argintar E, Berry M, Narvy SJ, Kramer J, Omid R, Itamura JM (2012). Hemiarthroplasty for the treatment of distal humerus fractures: short-term clinical results. Orthopedics.

[13] Burkhart KJ, Muller LP, Schwarz C, Mattyasovszky SG, Rommens PM (2010). Treatment of the complex intraarticular fracture of the distal humerus with the latitude elbow prosthesis. Oper Orthop Traumatol.

[14] Burkhart KJ, Nijs S, Mattyasovszky SG, Wouters R, Gruszka D, Nowak TE, Rommens PM, Muller LP (2011). Distal humerus hemiarthroplasty of the elbow for comminuted distal humeral fractures in the elderly patient. J Trauma.

[15] Parsons M, O’Brien RJ, Hughes JS (2005). Elbow hemiarthroplasty for acute and salvage reconstruction of intra-articular distal humerus fractures. Tech Shoulder Elbow Surg.

[16] Hohman DW, Nodzo SR, Qvick LM, Duquin TR, Paterson PP (2014). Hemiarthroplasty of the distal humerus for acute and chronic complex intra-articular injuries. J Shoulder Elbow Surg.

[17] Smith GC, Hughes JS (2013). Unreconstructable acute distal humeral fractures and their sequelae treated with distal humeral hemiarthroplasty: a two-year to eleven-year follow-up. J Shoulder Elbow Surg.

[18] Morrey BF, Chao EY, Hui FC (1979). Biomechanical study of the elbow following excision of the radial head. J Bone Joint Surg Am.

[19] Turchin DC, Beaton DE, Richards RR (1998). Validity of observer-based aggregate scoring systems as descriptors of elbow pain, function, and disability. J Bone Joint Surg Am.

[20] Brooker AF, Bowerman JW, Robinson RA, Riley LH (1973). Ectopic ossification following total hip replacement. Incidence and a method of classification. J Bone Joint Surg Am.

